# Antiviral Innate Immune Responses in Autoimmunity: Receptors, Pathways, and Therapeutic Targeting

**DOI:** 10.3390/biomedicines10112820

**Published:** 2022-11-04

**Authors:** Eirini Maria Stergioti, Theodora Manolakou, Dimitrios T. Boumpas, Aggelos Banos

**Affiliations:** 1 Laboratory of Autoimmunity and Inflammation, Center of Clinical, Experimental Surgery and Translational Research, Biomedical Research Foundation Academy of Athens, 115 27 Athens, Greece; 2 School of Medicine, National and Kapodistrian University of Athens, 115 27 Athens, Greece; 3 4th Department of Internal Medicine, Attikon University Hospital, National and Kapodistrian University of Athens Medical School, 124 62 Athens, Greece

**Keywords:** viral infection, autoimmunity, innate immunity, antiviral response, monocytes, macrophages, dendritic cells, NK cells, neutrophils, therapeutic opportunities

## Abstract

Innate immune receptors sense nucleic acids derived from viral pathogens or self-constituents and initiate an immune response, which involves, among other things, the secretion of cytokines including interferon (IFN) and the activation of *IFN-stimulated genes* (*ISGs*). This robust and well-coordinated immune response is mediated by the innate immune cells and is critical to preserving and restoring homeostasis. Like an antiviral response, during an autoimmune disease, aberrations of immune tolerance promote inflammatory responses to self-components, such as nucleic acids and immune complexes (ICs), leading to the secretion of cytokines, inflammation, and tissue damage. The aberrant immune response within the inflammatory milieu of the autoimmune diseases may lead to defective viral responses, predispose to autoimmunity, or precipitate a flare of an existing autoimmune disease. Herein, we review the literature on the crosstalk between innate antiviral immune responses and autoimmune responses and discuss the pitfalls and challenges regarding the therapeutic targeting of the mechanisms involved.

## 1. Introduction

The innate immune system provides an immediate defense mechanism by recognizing molecular structures produced by microbial pathogens and allows the adaptive immune responses to mediate an antigen-specific response. The initial sensing of a virus infection depends on the detection of molecules derived from pathogens by cellular receptors of innate immune cells that are encoded by inherited genes (germline-encoded host sensors) and called pattern recognition receptors (PRRs) [1,2], having a critical role in the host defense against viral particles. PRRs detect two classes of molecules: a. pathogen-associated molecular patterns (PAMPs), which are small molecular nonself motifs, such as viral nucleic acids, DNA, or RNA, and b. damage-associated molecular patterns (DAMPs), which are produced by or released from damaged or dying cells. PRRs that recognize viral PAMPs consist of the Toll-like receptors (TLRs), RIG-I-like receptors (RLRs), C-type lectin-like receptors (CLRs), and DNA sensors, such as IFI16 and the cGAS–STING signaling pathway [3,4,5]. The binding of PAMPs to PRRs triggers the activation of several signaling cascades in the host immune cells that lead to the expression of proinflammatory cytokines, type I interferons (type I IFNs), and *interferon-stimulated genes* (*ISGs*), to orchestrate the antiviral response and promote inflammation. The infection activates a robust and fine-tuned immune response that is crucial for the clearance of the virus. Short-term activation of the innate immune system is beneficial for host defense mechanisms, while overactivation of PRRs or downstream components may lead to a sustained immune system response and irreversible changes in organ structure and function [6,7,8,9,10]. The breakdown of immune regulatory mechanisms may culminate in the loss of self-tolerance, leading to an immune-mediated attack directed against both viral and self-antigens.

In autoimmune diseases, the imbalance between innate and adaptive immune responses may lead to hyperinflammation. Moreover, the exaggerated response of the immune cells with a hyperproduction of cytokines called a “cytokine storm” plays an important role in the manifestation and progression of autoimmunity. Cytokine storms occur in various autoimmune diseases, though the presence of a viral infection often serves as a trigger [11]. Cytokine storms are characterized by the hyperproduction of proinflammatory cytokines in response to various triggering stimuli (e.g., viral infection) leading the immune system to cause tissue damage. This aberrant immune response in autoimmunity in combination with a viral infection may lead to defective antiviral immunity or precipitate a disease flare. Herein, we review the literature on how antiviral mechanisms may drive autoimmune disease pathogenesis. Specifically, we report PRR-driven responses of innate immune cells that are involved in autoimmunity and discuss the pitfalls and challenges regarding the therapeutic targeting of the mechanisms involved.

### 1.1. Innate Detection of Viral Infection by PRRs

#### 1.1.1. Toll-like Receptors (TLRs)

TLRs serve as the first-line defense mechanism of the host in order to trigger the innate immune response and then orchestrate the initiation of the adaptive immune response [12]. The TLRs are type I integral membrane glycoproteins that contain leucine-rich repeats (LRRs) flanked by characteristic cysteine-rich motifs (involved in ligand binding) in their extracellular domain (ECD), a middle transmembrane domain (TM), and a C-terminus cytoplasmic Toll/IL-1 receptor (TIR) homology domain, which is essential for signaling. The formation of an M-shaped dimer or multimer is essential for the activation of all TLRs, so that the C-terminus regions of the two TLR ECDs are in proximity. The next step is the multimerization of cytoplasmic tails, which will then recruit the downstream adaptors Toll-interleukin-1 receptor (TIR) domain-containing adapter-inducing *IFN-β* (TRIF) or myeloid differentiation primary response 88 (MyD88) through homotypic interaction leading to the activation of specific transcription factors and inducing an antiviral type I interferon response and cytokine production.

**Figure 1:**
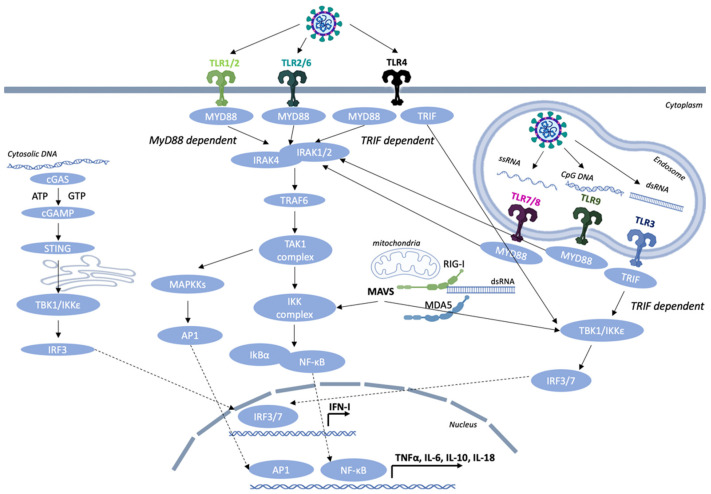
Innate immune cells sense viruses using distinct pattern recognition receptors (PRRs). Surface Toll-like receptors (TLRs) and TLRs located in endosomes, cytosolic nucleic acid sensors, RLRs, and DNA sensors detect viral nucleic acids or viral proteins. Most viral proteins are either components of the capsid or the envelope of the virus. Viral envelope glycoproteins are sensed via surface TLRs, such as TLR1/2, TLR2/6, and TLR4. Homo- or heterodimer formation initiates signaling to the two major downstream adaptor molecules, myeloid differentiation primary response 88 (MyD88) and Toll-interleukin-1 receptor (TIR) domain-containing adapter-inducing *IFN-β* (TRIF). Downstream signaling from surface TLRs requires the MyD88-dependent pathway. In endosomes, TLR3 detects double-stranded RNA (dsRNA); TLR7/8 detects single-stranded RNA (ssRNA), while TLR9 detects CpG DNA. TLR7/8 and TLR9 recruit the signaling adaptor molecule MyD88 to activate the IκB kinase (IKK) complex, resulting in the activation of nuclear factor kappa-light-chain-enhancer of activated B cell (NF-κB) and interferon regulatory factor (IRF) family members. In contrast, TLR3 binds with TRIF in order to activate downstream signaling, resulting in IRF3/7 translocation to the nucleus. The cytosolic sensors, retinoic acid-inducible gene I (RIG-I) and melanoma differentiation-associated gene 5 (MDA-5), sense viral dsRNA, and signal transduction occurs through the adaptor molecule mitochondrial antiviral-signaling protein (MAVS) located at the mitochondria that activates the TANK-binding kinase-1 (TBK1) and IκB kinase ε (IKKε) complex resulting in activation of NF-κB and IRFs. Cytoplasmic DNA is sensed through cytosolic DNA sensor cyclic GMP–AMP synthase (cGAS), which synthesizes cyclic guanosine monophosphate-adenosine monophosphate (cGAMP) in order to induce the ER-resident stimulator of interferon genes (STING) and leads to the activation of downstream molecules through the TBK1/IKKε complex. When NF-κB and IRFs are activated, they translocate to the nucleus and trigger the expression of proinflammatory cytokines and type I interferon (IFN) production. Secretion of these proteins promotes *interferon-stimulated genes* (*ISGs*) production, which results in the establishment of the “antiviral state”, recruitment of innate immune cells to sites of infection, and activation of the adaptive immunity to shape the overall antiviral immune response.

The cellular localization of TLRs correlates with their functions in sensing invading pathogens [13]. To date, 10 human TLRs (TLR 1–10) and 12 mouse TLRs (TLR 1–9, 11–13) have been identified, each one of them having a unique ligand specificity [14]. They collectively sense a wide range of bacteria, viruses, fungi, and endogenous ligands. In the current review, we focus on the TLRs that detect viral PAMPs. TLRs, based on their cellular localization, respond to different types of molecules. TLRs 3, 7, 8, and 9 are mainly expressed inside cells on the endoplasmic reticulum (ER) and endosomal membranes where they detect different viral nucleic acids [15]. TLRs found on the cell surface, such as TLR1, TLR2, TLR4, and TLR6, are able to mediate innate immune responses to viral pathogens or more specifically viral envelope and capsid proteins (Figure 1).

**TLR2** is expressed in various immune cells including neutrophils, monocytes/macrophages (MΦs), and dendritic cells (DCs). TLR2 detects a variety of microbial components, such as lipoproteins, peptidoglycan, and lipoteichoic acids, derived from Gram-positive bacteria, viruses, and parasites. Recognition of specific ligands by the host immune cells and the downstream signaling from TLR2 occurs with the heterodimerization with either TLR1 or TLR6. TLR1 and TLR6 contain highly homologous structures to TLR2 and are also expressed on the plasma membrane of all innate immune cells, such as monocytes, macrophages, and DCs [16]. ΤLR2/1 heterodimers mainly recognize bacterial triacylated lipopeptides, while TLR2/6 heterodimers tend to interact with mycoplasmal diacylated lipopeptides [17]. This ligand-mediated dimerization is important for the recruitment of the adaptor proteins, which are crucial for transmitting the signal to downstream effector molecules leading to proinflammatory cytokine production and the activation of an innate immune response. Although these heterodimers are best known for recognizing bacterial components, studies in mice have revealed that TLR2 contributes to the antiviral response in murine cytomegalovirus (MCMV), respiratory syncytial virus (RSV), and vaccinia virus infections [18]. More specifically, in mice with an RSV infection, the association of TLR2 with TLR6 in leukocytes mediates an immune response with the secretion of various costimulatory molecules, such as tumor necrosis factor alpha (TNF-α), interleukin 6 (IL-6), chemokine (C–-C motif) ligand 2 (CCL2), and chemokine (C–C motif) ligand 5 (CCL5) [19]. Moreover, TLR2 senses virus envelope proteins and glycoproteins in order to mediate antiviral immunity [20,21,22]. For example, the glycoproteins B and H from the cytomegalovirus (CMV) are detected by TLR2, resulting in nuclear factor kappa-light-chain-enhancer of activated B cell (NF-κB) activation in activated B cells and the production of proinflammatory cytokines [23]. In addition, the activation of TLR2 in Ly6C^high^ “inflammatory” mouse monocytes leads to the production of type I IFNs and the blocking of viral replication [24].

**TLR3** is expressed in myeloid DCs and macrophages and is not found in neutrophils and plasmacytoid dendritic cells (pDCs) and is localized in the endosomes [3]. TLR3 is a sensor of viral double-stranded RNA (dsRNA) and its synthetic analogue, polyinosinic:polycytidylic acid (poly I:C). TLR3 can also sense the presence of viral genomes derived from damaged host cells and viral particles, such as ssRNA and DNA viruses [22,25,26,27]. TLR3, in contrast to all other known TLRs, upon activation with synthetic or viral dsRNAs, does not recruit the adaptor molecule MyD88 and instead associates with TRIF [28]. TRIF binds to TNF receptor-associated factor 6 (TRAF6) and the receptor-interacting protein-1 (RIP-1) in order to favor the induction of NF-κB and MAPKs via TAK1 in a similar manner to MyD88. In addition, TRIF associates with TNF receptor-associated factor 3 (TRAF3) and binds TANK-binding kinase-1 (TBK1) and IκB kinase ε (IKKε) to favor the production of type I interferon by phosphorylating the interferon regulatory factor 3 (IRF3) and interferon regulatory factor 7 (IRF7). This allows their dimerization and entrance to the nucleus where they associate with NF-κB and activator protein 1 (AP-1) in order to transcriptionally activate inflammatory genes [29].

**TLR4**-expressing cells are mainly myeloid cells, such as monocytes, macrophages, and dendritic cells. TLR4 is an important PRR for Gram-negative bacterial components, such as lipopolysaccharide (LPS). Most myeloid cells express high levels of CD14, which facilitates the activation of the TLR4/MD2 complex by LPS. TLR4 association with myeloid differentiation 2 (MD2) on the cell surface is crucial for the activation of downstream adaptor proteins MyD88 and TRIF resulting in the expression of proinflammatory cytokines. CD14 also controls the immunoreceptor tyrosine-based activation motif (ITAM)-mediated tyrosine kinase Syk and its effector molecule phospholipase C gamma 2 (PLCγ2) to promote endocytosis and favor TLR4 internalization into endosomes for the activation of the TRIF-dependent signaling cascade [30]. It has also been revealed that TLR4 is an important sensor in the detection of endogenous molecules, such as DAMPs, released by inflamed tissues and necrotic cells [31]. In addition, TLR4 can also detect several viral glycoproteins that are found on the surface of an enveloped virus and mediate attachment with the target host cell by interacting with a cellular receptor and then fusion with the host membrane through the hydrophobic peptide. TLR4 mediates the production of IL-6 through the F protein upon RSV infection [32,33,34]. Moreover, TLR4 senses the envelope proteins of mouse mammary tumor virus (MMTV) and promotes the maturation of bone marrow-derived dendritic cells (BMDCs) by increasing the production of inflammatory cytokines, such as TNF-α, IL-6, and interleukin-12 subunit p40 (IL-12p40). Contrarily, in bone marrow-derived dendritic cells (BMDCs) upon MMTV infection, TLR4 enhances the expression of the MMTV entry receptor CD71 on these cells thus promoting viral infection [35,36]. Like viral glycoproteins, cellular glycoproteins are also detected by the immune system, potentially leading to autoimmune disorders. For instance, YKL-40, also known as chitinase 3-like 1 glycoprotein, is a member of chitinase-like glycoproteins and is produced in inflammatory conditions by neutrophils and macrophages. In joint tissues of rheumatoid arthritis (RA) patients, this glycoprotein is recognized as a potential biomarker of disease activity [37]. Moreover, dickkopf-related protein 1 (DKK-1) is another glycoprotein that plays a significant role in the inhibition of Wnt/b-catenin signaling by binding to the low-density lipoprotein receptor-related protein 5/6 (LRP-5/6) complex and favoring its degradation. DKK-1 is considered as a potential target for diseases associated with enhanced Wnt signaling activity. For instance, DKK-1 is elevated in the sera and urine samples of systemic lupus erythematosus (SLE) patients, and it is used as a positive biomarker for the identification of active lupus nephritis patients [38].

After ligand recognition by TLR4, the activation of two distinct signaling pathways are triggered, the MyD88-dependent and the MyD88-independent/TRIF-dependent pathways. TIRAP, which is a Toll-interleukin-1 receptor domain-containing adapter protein, mediates the signal from TLR4 to MyD88, whereas TRIF-related adaptor molecule (TRAM) mediates the signal from TLR4 to TRIF. A balanced activation between the MyD88- and TRIF-dependent pathways is crucial in order to elicit specific antiviral responses for controlling tumor cell growth and autoimmune diseases. A recent study by Mlcochova et al. [39] revealed that upon HIV-1 infection in macrophages, TLR4 binds to TRIF and induces G0 arrest and SAMHD1 antiretroviral activity by a MyD88/NF-κB-independent pathway.

**TLR7 and TLR8** recognize single-stranded RNA (ssRNA) molecules. TLR7 is predominantly expressed in plasmacytoid dendritic cells (pDCs) and monocytes and is involved in the induction of IFN-α gene transcription. On the other hand, human TLR8 is expressed in monocytes, macrophages, and conventional dendritic cells (cDCs) and in low-levels in B cells and pDCs [40,41,42]. Both receptor genes are located on the X chromosome, encode proteins that recognize self-RNA-containing autoantigens, and induce the production of IFN-α. The *TLR7* gene escapes X chromosome inactivation, and that may contribute to stronger female antiviral immunity and the female predisposition to SLE pathogenesis since IFN stimulation by TLR7 is a fundamental driver of SLE pathogenesis [43,44]. TLR7 and TLR8 also sense synthetic oligoribonucleotides (ORNs), such as imiquimod (R837), resiquimod (R848), and guanine analogue. TLR7/8 agonists have also been used as vaccine adjuvants due to their beneficial properties in host defense [39,40]. Upon ligand activation of TLR7 and TLR8, the dimer conformation changes, and the cytoplasmic TIR domains multimerize in order to recruit the downstream adaptor molecule MyD88 through homotypic interaction. MyD88 contains a C-terminus TIR (Toll IL-1R) domain for association with other receptors or adaptor molecules that contain a TIR domain and a N-terminus death domain (DD) for interacting with the interleukin-1 receptor-associated kinase (IRAK) family members. The association of MyD88 and TLR through their TIR domains results in the activation of the IRAKs, IRAK-1, and IRAK-4. In turn, IRAK-4 phosphorylates IRAK-1, which allows the binding to the C-portion of TRAF6 and enables the dissociation from the TLR complex. Upon activation, TRAF6 performs K63-linked polyubiquitination of the tumor growth factor beta (TGF-β)-activated kinase 1 (TAK1) and IκB kinase gamma (IKKγ), also known as NEMO (NF-κB essential modulator). IKKγ then interacts with the TAK1-binding protein 1 (TAB1), TAB2, and TAB3, resulting in IKK-mediated phosphorylation and the degradation of IκB. NF-κB is now able to translocate to the nucleus and induce gene transcription. Moreover, TAK1 forms a complex with TAB1, TAB2, and TAB3 that triggers the MAPK pathway leading to the formation of the activator protein 1 (AP-1) and its translocation to the nucleus. AP-1 and NF-κB are key modulators to orchestrate the expression of many proinflammatory genes. The formation of a complex by IRAK4, IRAK1, TRAF6, TRAF3, and the downstream transcription factors NF-κB and IRF7 leads to the activation and induction of proinflammatory cytokines and IFNs [44,45]. The complex of IRAKs and TRAF6 also associates with IRF5 and IRF7, leading to the subsequent IRAK1-dependent phosphorylation and nuclear translocation of both transcription factors. IRF5 is a key transcription factor in the induction of proinflammatory cytokines, such as IL-6 and IL-12p40, while IRF7 is primarily involved in type I IFN production [46,47]. A recent study by Marcken et al. [48] in human blood CD14^+^ classical monocytes infected with different RNA viruses, such as coxsackie (CV), encephalomyocarditis (EMCV), influenza A (IAV), measles (MV), Sendai (SV), or vesicular stomatitis virus (VSV), revealed that the RNA virus infection triggered distinct responses in the human monocytes, and the engagement of TLR7 or TLR8 is virus-specific. In detail, TLR7 favors the production of cytokines involved in CD4^+^ T helper 17 (Th17) cell polarization (IL-1β, IL-6, and IL-23) after virus infection with MV and VSV, whereas TLR8 promotes the T_H_1-promoting cytokine response and type I IFN production after viral infection with ECMV. On the contrary, the influenza A virus promoted the secretion of both types of cytokines. They also revealed that FOS-like 1 (FOSL1), which is an AP-1 transcription factor subunit, was increased upon TLR7 stimulation resulting in a reduced secretion of T_H_1-type cytokines, such as IL-27 and TNF-α in monocytes [48]. Moreover, an enhanced Ca^2+^ flux was induced by TLR7 rather than TLR8 signaling leading to a blockade of type I IFN production suggesting a contradictory role between these two receptors. Overall, these studies suggest that TLR7 and TLR8 activate different signaling cascades in human monocytes during RNA virus infection with different phenotypes in antiviral immunity.

**TLR9** is the only known endosomal ssDNA sensor. This receptor preferentially binds single- or double-stranded DNA and unmethylated CpG motif-containing viral DNA, such as herpes simplex virus (HSV) type 1 and type 2, leading to the production of type I IFNs and an antiviral immune response. TLR9 along with TLR7 is highly expressed in pDCs, also known as type I IFN-producing DCs and B cells among the rest of the immune cells [12,18,49]. CpG oligodeoxynucleotides (ODNs) are short synthetic DNA molecules containing cytosine (“C”) and guanine (“G”) motifs linked with a phosphodiester bond. They are naturally occurring analogs derived from viral and bacterial DNA. In addition, CpG ODNs are used as vaccine adjuvants in order to enhance the function of professional antigen-presenting cells (APCs) and favor the generation of vaccine-specific immune responses. There are three structurally distinct categories of CpG ODN: CpG-A, CpG-B, and CpG-C. The sequence of CpG ODNs, the secondary domains, and the effect on other immune cells play an important role in this separation. CpG-A preferentially induces the production of type I IFNs from pDCs and the maturation of APCs, but low B-cell stimulation. CpG-B favors strong activation of the B cells, induction of the TLR9-dependent NF-κB signaling cascade, and weakly stimulates type I IFNs and maturation of APCs. CpG-C combines functions of both classes as they strongly activate the secretion of IFN-α from pDCs, and they also stimulate B cells. TLR9 is located in the ER, and upon activation with CpG-DNA, it interacts with Unc93b and translocates to the endosomal compartments resulting in optimal TLR9 signaling [50]. In the endosome, ligand binding induces conformational change and the dimerization of TLR9, which results in the recruitment of the signaling adaptor molecule MyD88 [51]. The interaction between the TIR domains of MyD88 and TLR9 activates the IRAK4 and IRAK1. IRAK-4 is an essential modulator for the gene transcription of proinflammatory cytokines upon TLR9-induced activation of the signaling cascade. The activation of IRAK4 results in the recruitment of TRAF6, which in turn leads to the activation of TAK1. The phosphorylation of the IκB kinase (IKK) complex by TAK1 leads to the activation of NF-κB, MAPKs, and AP-1. The key transcription molecules NF-κB and AP-1 are then responsible for the induction of cytokines, such as IL-1, IL-12, and TNF, and the upregulation of costimulatory molecules, such as CD80 and CD86 [52]. Depending on the functional and morphological differences, endosomes can be classified as early or late. More specifically in pDCs, in the early endosomes, a signaling complex including IRAK4, IRAK1, TRAF6, and TRAF3 is formed, resulting in IRF7 activation and type I IFN production. In contrast, in the late endosomes of pDCs, the signaling complex includes IRAK4, TRAF6, TAK1, NF-κB, MAPKs, and IRF5, leading to the production of proinflammatory cytokines [53].

Failure to restore homeostasis by the uncontrolled expression of inflammatory mediators may predispose the host to autoimmune diseases, such as SLE and RA. To this end, fine-tuning of the TLR signaling cascades is pivotal in order to obtain a balance between pro- and anti-inflammatory immune responses for eliminating invading pathogens without damaging the host. Several regulatory checkpoints in the TLR pathways are developed in order to tightly regulate the immune system’s response including (i) removal of receptors from the cell surface, (ii) expression of negative regulators of the signaling cascades, (iii) adaptor complex destabilization, (iv) phosphorylation and ubiquitin–proteasome-mediated control of the signaling molecules, (v) manipulation of the expression of the other receptors and downstream components, and (vi) transcriptional control [54,55]. Potentially harmful TLR signaling pathways can be regulated by negative feedback mechanisms and by anti-inflammatory factors, such as interleukin (IL)-10 and steroids [56,57]. A study by Curtale et al. [57] revealed an increased expression of miR-146b upon LPS stimulation through an IL-10-dependent loop. They also highlighted that this miRNA exhibited anti-inflammatory features in human monocytes by targeting TLR4 and several other components of the TLR4 signaling cascade, such as MyD88, IRAK-1, and TRAF6, thus suggesting that miR-146b is a negative modulator of the TLR-induced inflammatory response. In addition, many combinations of TLR-TLR and TLR-NOD modulate inflammatory responses. For instance, the NOD-like receptor family CARD domain containing 3 (NLRC3) regulates the activation of the transcription factor NF-κB upon TLR stimulation by inhibiting the TRAF6 activation. In a study by Schneider et al. [58], the expression levels of NLRC3 were reduced upon LPS stimulation, and mice lacking the NLRC3 developed enhanced secretion of proinflammatory mediators, proposing a negative role of NLRC3 in the TLR signaling cascade.

#### 1.1.2. RIG-like Receptors (RLRs)

**RIG-like receptors (RLRs)** are cytoplasmic sensors of viral infection and key players in the recognition of viral nucleic acids by inducing the secretion of type I IFNs and chemokines. RLRs can sense double-stranded RNA and DNA/RNA heteroduplex oligonucleotides, including regions of the genome of RNA viruses and RNA transcripts of RNA and DNA viruses. The two best-characterized RLRs are the retinoic acid-inducible gene I, **RIG-I**, and the melanoma differentiation-associated gene 5, **MDA5**. RLRs are characterized by a conserved domain of a central DExD/H-box helicase region and a C-terminus domain (CTD), both of which are implicated in the recognition of viral RNA. In addition, both RLRs contain two N-terminus caspase activation and recruitment domains (CARDs), and upon sensing of viral components, they induce the activation of downstream signaling molecules resulting in type I IFN production [59]. More specifically, RIG-I and MDA5 detect distinct types of viral dsRNAs. RIG-I senses short dsRNA (<1000 bp) and a 5′ triphosphate (5′ ppp) moiety, found in the genomic RNA of several viruses, in association with short blunt-end double-stranded RNA (dsRNA), such as “panhandle” domains, that are important for RIG-I’s ability to discriminate viral from self-RNA. Host cell RNA evades recognition by RIG-I due to post-transcriptional modifications, such as 5′ppp capping with 7-methyl guanosine (m7G) and 2′-0-methylation of 5′-end nucleotides [60,61]. MDA5 recognizes long-chain dsRNA fragments (at least 2 kbp) organized in web-like structures [62]. Mitochondrial antiviral-signaling protein (MAVS) acts as a central hub for signal transduction initiated by RIG-I and MDA5 via TBK1 and IKKε in order to activate NF-κB and IRFs, leading to the expression of proinflammatory cytokines and type I interferons [63]. RIG-like receptors are expressed in a wide variety of cell types, including bone marrow-derived leukocytes and various tissue cells, and enable them to participate in innate immune responses to these viruses.

#### 1.1.3. The cGAS–STING Pathway

**Stimulator of Interferon Genes (STING)** is a protein consisting of four transmembrane regions (TMs) and a CTD and is located in the ER. Human STING contains a transmembrane domain in the N-terminus for the regulation of its cellular localization and homodimerization and an intracellular soluble portion in the C-terminus for interacting with downstream molecules, including TBK1/IKKε and IRF3/IRF7. Upon recognition of cytoplasmic DNA from DNA viruses and abnormal endogenous DNA, the **cytosolic DNA sensor cyclic GMP–AMP synthase (cGAS)** synthesizes cyclic guanosine monophosphate-adenosine monophosphate (2′,3′-cGAMP) in order to induce translocation of the ER-resident adaptor protein STING to the endoplasmic reticulum–Golgi intermediate compartment (ERGIC) and then the Golgi apparatus and the endosomes for degradation in lysosomes. The activated STING dimer recruits TBK1 to form the translocation complex from the ER to the perinuclear lysosomal compartments through an autophagy-like process. The STING–TBK1 complex phosphorylates IRF3 and NF-κB to promote entry into the nucleus. Then, IRF3 and NF-κB induce the production of *type I IFN* genes and other proinflammatory cytokines through the TBK1–IRF3 axis and NF-κB signaling pathway, establishing an antiviral state [64,65,66].

## 2. Viral Infection and Autoimmunity

**Figure 2:**
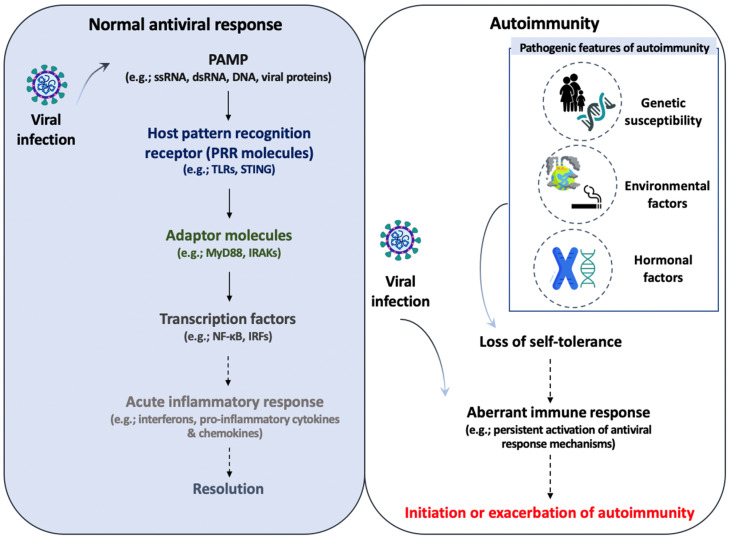
PRR signaling upon viral infection may either lead to acute inflammatory response and resolution or trigger autoimmune responses. Innate immunity is activated upon viral exposure, and the response is mediated through pattern recognition receptor (PRR) molecules that recognize the viral nucleic acids, the adaptor molecules that mediate the signal to downstream components, and the transcription factors that are responsible for the outcome. An acute inflammatory response is orchestrated by the release of antiviral molecules, such as interferons, proinflammatory cytokines, and chemokines at sites of infection. In autoimmune diseases, a combination of genetic susceptibility, such as gene copy variations and single-nucleotide polymorphisms (SNPs) and environmental as well as hormonal factors including UV light, toxic chemicals, and genes defined by the X chromosome, leads to loss of self-tolerance and chronic inflammation. In this environment, when a viral infection occurs, the host defense mechanisms are exposed and may promote an exaggerated immune response, which can lead to initiation or exacerbation of autoimmunity.

Immunological tolerance is an active, tightly regulated, fine-tuned response of the immune system to self-antigens or against various environmental entities that prevent the immune system to mount possibly harmful responses. There are two types of immune tolerance, central and peripheral tolerance, and both provide and maintain self-tolerance. The discrimination between self- and nonself antigens is pivotal for the proper functioning of the immune system. Failure of immunological tolerance leading to an aberrant immune response against host antigens is critical for the development of autoimmunity [67]. Several triggering factors have been linked to autoimmune responses, such as genetics, environment, age, and viral infections. Viral infection can alter immunological tolerance against self-antigens and has been associated with the initiating or flaring of several autoimmune and inflammatory phenomena in individuals with genetic susceptibilities [68,69,70,71]. These infections trigger the antiviral immune response mechanisms resulting in the activation of signaling pathways and the induction of cytokine and chemokine production, the production of autoantibodies, and the deposition of immune complexes (ICs) in tissues some of which could overwhelm the immune regulatory mechanisms. Several mechanisms, such as molecular mimicry, bystander activation of dendritic cells and T-cells, and epitope spreading can explain how viruses might trigger a series of actions leading to the development of an autoimmune disease [68,72]. For instance, the possibility that Epstein–Barr virus (EBV) may trigger several autoimmune diseases, such as SLE and multiple sclerosis (MS), has been reported during the past decades [73,74]. Several studies have demonstrated an increased viral load of EBV DNA in SLE patients compared to healthy individuals [75,76]. Furthermore, a serologic association to EBV infection has been reported with high titers of anti-early antigen (EA) IgG and IgA in SLE patients compared to healthy controls [73,77]. In addition, several reports have revealed the molecular similarity between the EBV nuclear antigen-1 (EBNA-1) and the common lupus autoantigen Ro, as well as the inability of CD8^+^ T cells to control EBV-infected B cells suggesting that viruses may influence the development of SLE pathogenesis [78,79,80,81,82]. Therefore, environmental factors, such as viral infections, may influence the function of PRRs and the expression of downstream molecules involved in the signaling cascades. In innate immune cells, PRR signaling upon viral infection may exaggerate immune responses in autoimmunity (Figure 2). How risk alleles in PRR signaling pathways and genetics contribute to autoimmunity and how viral-induced autoimmunity can be carried out through the above-mentioned mechanisms, is outside the scope of the current review and is discussed elsewhere [68,72,83,84,85,86,87]. However, in the current review, we focus on the crosstalk between innate antiviral immune responses and autoimmune responses mediated by PRR molecules and downstream components and discuss the potential therapeutic targeting of the mechanisms involved.

### 2.1. The Role of Innate Immune Cells in Antiviral Responses in an Autoimmune Background

#### 2.1.1. Linking Plasmacytoid Dendritic Cell (pDC) Antiviral Response with Autoimmunity

Plasmacytoid dendritic cells (pDCs), members of the dendritic cell (DC) family, are key players in antiviral immunity and are known to secrete large amounts of type I IFNs in response to viremia. They represent a heterogeneous cell population with 0.2–0.8% of peripheral blood mononuclear cells (PBMCs) that links innate and adaptive immune responses [88]. The main function of these cells is to detect viral nucleic acids through TLR7 and TLR9; capture, process, and present antigens to adaptive immune cells; and mediate their polarization into effector cells orchestrating a proper immune response [89,90]. TLR7- and TLR9-mediated PRR signaling in pDCs has been reported in various autoimmune conditions suggesting their role in the aberrant immune response, such as cytokine storm and excessive activation of the innate and adaptive immune system observed in many autoimmune and inflammatory phenomena. The emerging literature indicates how type I IFN produced by pDCs in antiviral immunity may contribute to autoimmune pathology and how similar pathways are triggered and may drive disease pathogenesis [91,92,93,94].

Hillen et al. [95] demonstrated that in primary Sjögren’s Syndrome (pSS), which is a systemic autoimmune disease characterized by salivary and lacrimal gland dysfunction, circulating pDCs from pSS patients display an activated transcriptional profile and are primed for enhanced proinflammatory cytokine secretion compared to healthy donor (HD) pDCs. More specifically, they exhibit high levels of proinflammatory cytokines upon stimulation with endosomal TLR ligands as the activation of TLR7 triggered more type I IFN production in pSS pDCs compared to HD, similar to an antiviral response. Moreover, in a study by Mavragani et al. [96], endogenous virus-like genomic repeat elements in pSS patients triggered the IFN-I pathway through the activation of TLR7/8 signaling cascade in pDCs further influencing the initiation or amplification of SS. Thus, such studies provide insights into the role of viral infection in the initiation or propagation of several autoimmune diseases. Another recent study by Wang et al. [97] revealed that TLR7 signaling influences the development of Sjögren’s syndrome (SS) since TLR8-deficient mice that develop SLE due to enhanced TLR7 signaling by DCs also develop a secondary pathology similar to SS. This highlights that the development of the SS phenotype is dependent on TLR7 signaling. In light of this, they also revealed an increased TLR7 expression and enhanced inflammatory cytokine and chemokine secretion, such as TNF, LT-α, CXCL13, and CXCR5 in pDCs of pSS patients. These data substantiate the role of TLR signaling in mediating the inflammatory features of pDCs in pSS immunopathology supporting their contribution in the initiation or progression of autoimmunity. This enhanced signaling in pSS pathology through TLR7/8, often triggered by viral entities, suggests that PRRs promote and influence the progression of autoimmune diseases by favoring a sustained inflammatory response.

Systemic lupus erythematosus (SLE) is a chronic multisystem autoimmune disease that manifests a wide range of clinical and molecular abnormalities and is characterized by the loss of self-tolerance to nuclear antigens [98]. An elevated expression of type I IFNs and *type I IFN*-regulated genes termed as the IFN signature has been reported in the majority of SLE patients [99,100,101,102]. The IFN gene signature observed in SLE patients is characterized by the increased expression of *IFN-regulated* genes, such as *ISG15*, *IFI16*, and *FcgRI* (*CD64*), and is mainly reflected by the circulating type I IFNs [101,103]. The disease activity correlates with *IFN-α* expression levels and the strength of the IFN signature [104,105,106]. In lupus, IFN-α-driven immunologic alterations culminate into persistent self-directed immune responses against autologous nucleic acids, mimicking a sustained antiviral response. In detail, an initial viral infection that can be sensed by different PRR molecules and mediated by several signaling cascades as discussed previously, can promote type I IFN secretion and release of cellular material from dying or apoptotic cells. Then, apoptotic cells release DNA- or RNA-containing autoantigens as well as neutrophil extracellular traps (NETs), which triggers B cells to produce autoantibodies against CpG-rich DNA or ss-RNA and associated proteins. The formation of interferogenic ICs will act as an endogenous adjuvant for triggering type I IFNs, leading to a prolonged activation of the immune system to produce IFNs. This will result in the chronic activation of the IFN system, which will enable the development of autoimmune phenomena, chronic inflammatory processes, and tissue damage in a vicious circle [107].

IFN-α has multiple immunostimulatory properties that include the expression of several pivotal molecules in the response of the immune system, such as MHC II, CXCL10, CXCR3, CD40, CD80, and CD86. Depending on the cell type exposed to IFN, the effects will vary and may be detrimental. For example, type I IFNs influence the function of B cells through several mechanisms, such as the production of B-cell activating factor (BAFF) from monocytes and neutrophils, thus leading to prolonged survival and activation of B cells and enhanced T-cell-independent and -dependent antibody production [108,109]. Genetic, epigenetic, environmental, and immunoregulatory factors influence the outcome of SLE pathogenesis (Figure 2) [110,111]. It is also well established that SLE is a female-predominant disease, although the causes of sex bias are ill-defined. Moreover, mounting evidence suggests that PRRs promote and influence the progression of autoimmune diseases by favoring a sustained inflammatory response to self-components [112,113]. In addition, enhanced TLR7-mediated IFN-α production was also demonstrated in pDCs from SLE patients in a study by Murayama et al. [114]. It was also reported that pDCs stimulated with the TLR7 agonist promoted an autoimmune Th17 phenotype and increased levels of type I IFNs were correlated with high amounts of Th17 cytokines in the serum of SLE patients [115,116]. In addition, a distinctive feature of SLE immunopathology is the increased numbers of ICs in the serum of SLE patients correlating with disease severity [117]. It was revealed that these nucleic acid-containing immune complexes are internalized by pDCs via FcγRIIa into endosomes where they stimulate TLR7 and TLR9 leading to type I IFN secretion. Also, type I IFN production by pDCs stimulated with ICs is robustly enhanced in the presence of activated T cells [118,119]. Furthermore, it is well established that EBV RNA and DNA enhance the secretion of IFN-α through TLR7 and TLR9 in pDCs and SLE patients demonstrate high titers of EBV and increased *latent membrane protein 1* (*LMP1*) expression levels, which is a well-known oncoprotein of the EBV latent gene products [114,120]. Thus, these studies highlight that EBV might be linked with the initiation and progression of SLE since the type I IFN pathway is activated. In addition to this, it has also been reported that IFN-α and several other proinflammatory cytokines are able to induce LMP-1 expression in B cells infected with EBV thus supporting the notion that EBV and its products are involved in key pathways modulating SLE activity and severity and may exacerbate the autoimmune phenotype observed via promoting IFN production through PRR pathways [121]. Interestingly, it seems that viruses can trigger autoimmunity through several mechanisms, one of them being the stimulation of intracellular PRR inflammatory cascades thus leading to the production of IFNs and cytokines that may excessively activate the immunoregulatory mechanisms and predispose to autoimmunity or exacerbate the pathology. Another study by Dominique et al. [122] on systemic sclerosis (SSc), which is a multisystem, fibrosing autoimmune disorder characterized by immune dysregulation, revealed that SSc pDCs demonstrated enhanced expression of TLR8 leading to the promotion of IFN-α production. This study underlines the key role of pDCs in the sensing of RNA and the subsequent activation of TLR inflammatory cascades in the establishment of fibrosis (Table 1). Moreover, a possible pathogenic association of viral infection and the development of SSc has been proposed, with the human parvovirus B19 (B19) and human cytomegalovirus (HCMV) as the important triggering agents in SSc pathology. Further studies are needed in order to expand our knowledge on the crosstalk between SSc viral products, PRR inflammatory pathways, and SSc immunopathology.

In summary, pDCs are important players in the mechanisms underlying several autoimmune diseases and demonstrate enriched inflammatory responses through the activation of TLR7/8 and TLR9 signaling pathways and the IFN system. Frequently, this activation is either triggered by environmental agents, such as viruses or self-components, both resulting in the excessive stimulation of the immune response. It is of great importance to evaluate how these cells can orchestrate an effective antiviral immune response with type I IFN production and cytokine secretion in autoimmunity.

#### 2.1.2. Linking Monocyte Antiviral Response with Autoimmunity

Monocytes are blood mononuclear cells that arise from bone marrow progenitors. Based on CD14 (lipopolysaccharide (LPS) coreceptor) and CD16 (FcγRIII) expression levels, human monocytes are divided into three phenotypically and functionally distinct populations: the CD14^++^CD16^−^ classical, CD14^++^CD16^+^ intermediate, and CD14^low^CD16^++^ nonclassical [123,124]. They are key players in recognizing pathogen-associated molecular patterns via PRRs and eliciting an immune response via the secretion of proinflammatory cytokines. By sensing the inflammatory environment, circulating monocytes can replenish the pool of tissue monocyte-derived macrophages (moMφs) and inflammatory monocyte-derived dendritic cells (moDCs) [125,126]. Several lines of evidence showed that monocyte activation is associated with the disease progression and severity in several autoimmune diseases [127]. Nucleic acid sensing leads to the activation of IFN-α immunity, which in combination with clearance pathways, orchestrates the antiviral response. However, how can the aberrant activation of the PRR signaling pathways in monocytes through the sensing of nucleic acids or other triggering factors be implicated in the development of autoimmunity?

A recent study by Kyogoku et al. [128] revealed that monocytes from SLE patients demonstrated a pathogenic IFN signature (activation of IRFs, GTPases, and kinases) [102] observed in autoimmune conditions augmented by the expression of cytokines, such as IL-9, IL-10, and IL-15 and mediated by the JAK/STAT signaling pathway. This can be compared to monocytes from HDs immunized with the yellow fever vaccine (YFV), which express more normal cell-specific and virus-induced signatures. A common IFN signature is a pure virus-induced signature detected in healthy donors immunized with the influenza vaccine and has a composition of mainly type I IFNs, which are the major antiviral cytokines. In contrast, type I IFNs are also dominant in SLE pathogenesis and govern the immune responses of CD4^+^ T cells and monocytes, but at the same time, these responses are influenced by additional immunoregulatory events. Such events might be responsible for the difference observed between “common” and “autoimmune-specific” IFN signatures, reflecting the sustained IFN response in SLE patients. Moreover, the IFN signature observed in SLE patients leads circulating monocytes to differentiate into potent antigen-presenting dendritic cells (DCs) with an increased capacity to orchestrate T and B cell responses [129]. In a study by Gkritzimanaki et al. [130], mitochondrial DNA (mtDNA) was accumulated in the cytosol of CD14^+^ monocytes due to a deregulation of the mitochondrial metabolism caused by IFN-α and the dysfunction of autophagic digestion and was sensed via STING to favor the differentiation to autoinflammatory DCs and enhance the autoimmune response. Specifically, autoinflammatory DCs activate T cells and then contribute to the expansion and survival of autoantibody-producing cells by T–B cell aberrant communication [131]. STING and one of its ligands, mtDNA, cause an immune stimulatory output through NF-κB and/or TBK1/IRF3 similar to an antiviral response and have a critical involvement in disease pathogenesis. Moreover, the overexpression of *TLR7* due to the duplication of the Yαα (Y-chromosome-linked autoimmune acceleration) locus leads to the exacerbation of autoimmunity in murine lupus [132]. In addition, a recent study by Brown et al. [133] showed that a *TLR7* gain of function gene variant may cause human SLE. In addition, another recent study by Murakami et al. [134] demonstrated that TLR7 drives autoantibody production and lupus-associated monocytosis in NZBWF1 mice. The antiTLR7 mAb, but not antiTLR9, ameliorated nephritis in lupus-prone mice by inhibiting IgG deposition in glomeruli and autoantibody production in B cells and monocytes. They also revealed that Ly6C^low^ patrolling monocytes were enhanced in the circulation, spleen, and glomeruli of NZBWF1 mice and displayed an overexpression of genes linked with lupus pathogenesis, such as *TLR7*, *IL-10*, *CD115*, *CD31*, and *TNFSF15*. This evidence suggests the importance of TLR7 in the progression of the disease as the hyperactivation of TLR7 may cause the uncontrolled sensing of several triggering components and an aberrant inflammatory response.

In addition, germline mutations in the human *SAMHD1* gene represent the main cause of the progression of the autoinflammatory Aicardi–Goutières Syndrome (AGS). SAMHD1 protein is known to restrict HIV-1 infection in cells of the myeloid lineage, such as monocytes/macrophages and DCs, by inhibiting the synthesis of viral DNA. AGS mimics congenital viral infection as defective nucleases in AGS are involved in the deficient removal of endogenous nucleic acids resulting in the accumulation of ssDNA and the chronic activation of the innate immune response and the DNA damage response network [135,136,137]. The phenotypic overlap of AGS with congenital infection and some traits of SLE pathogenesis highlights the IFN-α-mediated immune response upon viral and host nucleic acids triggering the PRR signaling cascades [138]. A recent study [139] revealed that SAMHD1 KO human monocytic THP-1 cells displayed nucleic acid deposition in the cytosol and spontaneous expressions of type I IFNs and *ISGs*, replicating the phenotype observed in patients with AGS. The inhibition of the TBK1–IRF3 pathway, through BX795, which is an inhibitor of the catalytic activity of TBK1/IKKε thus attenuating the phosphorylation of IRF3 and blocking its activation, overruled the secretion of type I IFNs observed in the SAMHD1 KO cells. Therefore, in AGS patients, monocytes exhibit a failure to process nucleic acids leading to the activation of autoimmune responses.

Rheumatoid arthritis (RA) is an autoimmune disease in which many cells from the innate and adaptive immune branch take part in the development of inflammation in synovial joints. Focusing on monocytes, these cells display a significant role in the progression of synovial inflammation since they are recruited at sites of infection by interacting with chemotactic ligands that are present in fibroblasts, such as synoviocytes (FLS), and other autoimmune cells, and sustain the perpetuation of autoimmunity [140]. The interplay between genetic and environmental factors as well as a defective immune response is of great importance in the development and progression of RA pathogenesis [141]. Furthermore, infection by viral particles, such as EBV, has been linked with multiple malfunctions in RA, increasing the prevalence of flares of disease activity [72]. It is well established that human B cells are the main targets of EBV, although several reports indicate that EBV can also infect other cells including monocytes [79,142,143]. In a study by Lacerte et al. [144], active RA patients demonstrated an overexpression of TLR2 and TLR9 in blood and synovial monocytic subsets and an increased secretion of a wide range of proinflammatory cytokines upon induction with synthetic and viral ligands for TLR2 and TLR9. They also demonstrated that the EBV genome was present in monocytes and neutrophils, strengthening its role in the exaggeration of the disease [144,145]. Another study revealed that the expression of TLR2 in CD16^+^ blood monocytes contributed to the production of TNF-α in RA patients [146]. The above-mentioned findings suggest that both classical and intermediate monocytes are key players in the development of inflammation in the tissues of active RA patients through the PRR-mediated activation of inflammatory cascades. In addition, classical monocytes produce costimulatory molecules and chemoattracting factors and regulate the progression of inflammation. Viral components, such as EBV, virions can induce the activation of TLR2 and TLR9 in the synovial compartment and sustain the inflammatory response, causing the exacerbation of the disease in susceptible RA patients. Moreover, the removal of pathogenic components through phagocytosis could also trigger TLR activation. Neutrophils and macrophages are the main phagocytes of the immune system as they engulf dead cells and then elicit an inflammatory response. Activation of the innate immune response may also occur, for instance, when the genetic material of dead cells is not effectively degraded, leading to B cell activation through B cell receptor (BCR) and TLR stimulation [147]. Complement deficiencies is another way of promoting autoimmune features in SLE through insufficient clearance [148]. This insufficient clearance may lead to the break of self-tolerance and then to autoimmunity [149].

Another study by Farina et al. [150] revealed that newly lytic EBV mRNA enhanced TLR8 expression in infected SSc and HD monocytes. MyD88 and IRF7 expression was also induced in infected EBV monocytes from SSc patients and HDs. This increase was associated with a robust induction of *IFN-regulated genes* and chemokines, such as CXCL9, OAS3, Siglec1, CCL2, IL-6, and TNFα, in SSc- and EBV-infected monocytes, which are markers associated with the activation state of monocytes. Studies with THP-1 cells indicated that EBV was influencing the immune system in a TLR8-dependent manner. Moreover, viral mRNA and proteins were detected in freshly isolated SSc monocytes where a microarray analysis demonstrated an increased IFN proinflammatory response and an altered level of TLR8 expression in EBV-infected SSc monocytes compared to HD monocytes. Overall, through the EBV paradigm, this study highlights that TLR8 possesses a central role in the activation of SSc monocytes by mediating a robust increase in the IFN signature. In addition, the activation of monocytes in SSc through TLR8 might be attributed to an EBV infection, thus supporting the notion that viral infection promotes autoimmunity through PRR signaling cascades, which affects the IFN innate immune responses.

Primary Sjögren’s Syndrome is also associated with the altered immune response of monocytes. Additional research by Lopes et al. [151] demonstrates that the transcriptome of pSS monocytes is enriched for gene expression profiles associated with intermediate and nonclassical monocytes. Monocytes from pSS patients exhibit an activating profile with dysregulation in gene expression for translation, IFN signaling, and TLR signaling pathways, such as TLR4, TLR5, and TLR7/8. Serum from pSS patients primed monocytes to an increased secretion of TNF-α upon activation with TLR ligands compared to HD monocytes, and that may promote inflammation and the activation of other immune cells and contribute to pSS immunopathology. As it has been previously shown [152], monocytes from pSS patients exhibit an impaired phagocytic capacity of apoptotic cells and fail to promote an immunosuppressant cytokine profile to resolve inflammation and tissue injury. The interferon-α/β receptor (IFNAR) is a cell surface receptor that binds type I IFNs leading to the activation of the JAK–STAT signaling and the MAPK, PI3K, and Akt signaling cascades and therefore favoring the production of *ISGs*. IFNAR blockade partially abrogated the alterations observed, suggesting that the transcriptome profile of pSS monocytes can be characterized as IFN-dependent and independent (Table 1). However, it remains to be established whether monocytes in pSS pathology are functional in order to mediate antiviral immune responses. In this direction, characterizing the mechanisms of actions mediating the sustained activation of monocytes in pSS seems to be of great importance.

Collectively, these studies highlight the activating profile of monocytes with aberrant PRR response in various autoimmune diseases. This abnormal PRR response is often triggered by viral components culminating in the initiation or exacerbation of autoimmunity. The exact causes for the observed PRR manifestations and the differential roles of PRR signaling in autoimmunity but also how the antiviral response is affected remain to be defined in more detail.

#### 2.1.3. Linking Macrophage Antiviral Response with Autoimmunity

Macrophages are tissue-resident or infiltrated immune cells with critical immunoregulatory, antimicrobial, and tissue-repairing roles to decrease immune reactions and promote tissue regeneration. They either originate from yolk sac (YS) progenitors during embryonic development and are maintained in postnatal life in certain adult tissues, or they derive from bone marrow (BM) hematopoietic stem cell (HSC) progenitors and circulating monocytes [125,153,154,155]. M1-like (classically activated) and M2-like (alternatively activated or wound-healing) macrophages are the two major subsets of activated macrophages with distinct cellular and molecular functions. Both subsets are involved in inflammatory responses with the difference that M1 macrophages have an important role in pro-inflammatory response, whereas M2 macrophages are mostly involved in tissue repair and anti-inflammatory responses [156,157]. Recent data have demonstrated that the activation of macrophages cannot be fully described using the M1/M2 paradigm. Macrophage phenotype alternates in response to various stimuli. For example, an increased number of macrophages in the circulation expresses both M1 surface markers, such as CD80, CD86, and TLR4 and M2 molecules, such as CD204 and CD163. In addition, macrophages express various TLRs, such as TLR2, TLR3, TLR4, and TLR7/8, and have many functions in restoring cell homeostasis. Some of their roles include the recognition and elimination of invading pathogens through phagocytosis and the subsequent presentation of antigens to T cells in order to orchestrate an inflammatory response [158]. There is also growing evidence supporting a causal link between the presence or activation of macrophages and the development of autoimmune diseases [159]. Their contribution to autoimmunity and inflammation comes through their ability to present autoantigens and their potent effector mechanisms, unleashed during innate and adaptive immune responses [159]. However, the emerging literature highlights a causal link between antiviral response mechanisms in macrophages and autoimmune phenomena [159,160,161,162].

IRF3-induced type I interferon production has an important role in antiviral responses and SLE. Serine/threonine kinase AKT2 regulates the type I IFN production by phosphorylating IRF3 at Thr207 to attenuate IRF3 nuclear translocation, resulting in diminished type I IFN production. To this end, a recent study by Zheng et al. [163] demonstrated that in viral-infected macrophages or monocytes and samples from SLE patients and mouse models, AKT2 expression is decreased, and *Akt2* deficiency promotes IFNβ1 and the production of *ISGs* to enhance an antiviral defense while heightening SLE in mouse models. These findings indicate that cells in autoimmune diseases, such as lupus, may be already prone to PRR defects, and the infection of viral pathogens further exacerbates the pre-existing deregulated PRR signaling [81,82,164,165,166,167].

Macrophages also play an important role in the progression of rheumatoid arthritis (RA). A study by Quero et al. [168] revealed that differentiated M2 macrophages from monocytes of HD or RA patients depicted an impeded anti-inflammatory profile due to the production of proinflammatory cytokines, such as IL-6 and IL-8, upon TLR2 stimulation. The critical role of TLR signaling in the pathogenesis of RA has been already stated by various studies in murine arthritis models. Abdollahi et al. [169] demonstrated that the development of streptococcal cell wall (SCW)-induced arthritis in mice was dependent on TLR2 during the acute phase, and this effect was changed to TLR4 dependency during the chronic joint inflammation phase. They have also previously reported that the inhibition of TLR4 by a TLR4 antagonist in a collagen-induced RA mouse model (CIA) repressed the clinical manifestations and the severity of arthritis [170]. Moreover, TLR7 displays high levels in synovial tissue (ST) lining and sublining macrophages (CD68^+^ macrophages identified in synovial biopsies) from RA patients. In a study by Kim et al. [171], it was revealed that miR-let-7b is a TLR7 endogenous ligand and is found in RA synovial fluid macrophages. The activation of TLR7 by miR-let-7b favors the differentiation of anti-inflammatory into inflammatory M1 macrophages and promotes the progression of arthritis. It was also shown [172] that ligands present in the inflamed joint of RA patients, stimulate TLR3 and TLR7 leading to a proinflammatory cytokine production in an IRF5-dependent manner. Therefore, since TLR signaling is enhanced in the macrophages of RA patients in a similar manner to an antiviral response, it would be intriguing to extend the investigation beyond the systemic autoimmune phenotype and delineate how nucleic acids and viral infection mediated by these TLRs would be affected.

Another recent study by Witas et al. [173] demonstrated that when C57BL/6 (B6) and Sjögren’s syndrome-susceptible (SS^S^) bone marrow-derived macrophages (BMDMs) were incubated with apoptotic cells (ACs), an inflammatory profile was induced in SS^S^ BMDMs. This profile was characterized by the overexpression of genes involved in the IFN signaling pathway, costimulatory molecules, and myeloid activation genes as well as inflammatory cytokines, such as IL-6, IL-12b, IL-1β, and IL-10. Witas et al. also demonstrated an increased *TLR7* and *TLR9* expression in PBMCs of pSS patients and increased secretion of IL-1β and TNF. By inhibiting TLR7 and TLR9, they showed a decreased inflammatory response of SS^S^ BMDMs to ACs. A separate study by Wang et al. [97] also indicated that TLR7 expression is enhanced in SS patient salivary gland tissue leading to the secretion of inflammatory cytokines and chemokines, such as TNF, CXCL12, and CXCR5. This group also reported that TLR8 KO mice developed SS pathology that was driven by enhanced TLR7 signaling. After the inhibition of *TLR7* and *TLR9*, a diminished AC-induced secretion of inflammatory genes in SS^S^ BMDMs was observed. This underlines that the inflammatory response observed upon AC activation in SS^S^ BMDMs is mediated by the stimulation of *TLR7* and *TLR9*. In addition, monocytes from SS patients that are IFN positive exhibit a high expression of *TLR7* and downstream effector molecules *MyD88*, *RSAD2*, *IRF7*, *RIG-I*, and *MDA5* [174]. To conclude, the AC stimulation of TLR9 resembles the inflammatory milieu in SS autoimmune disease as in many other autoimmune diseases, and the enhanced inflammatory cytokine and costimulatory response in SS^S^ BMDMs may contribute to the initiation of an autoimmune environment. In general, macrophages, as well as DCs, are efferocytic cells that can elicit a rapid and efficient clearance of apoptotic cells and debris in order to maintain cell homeostasis and sustain self-tolerance. Through efferocytosis, macrophages ensure the well-organized elimination of apoptotic cells without antigen presentation and inflammation. When efferocytosis is disturbed, apoptotic cells can rupture, secreting cellular components and inducing a hyperactivation of the innate and adaptive immunity resulting in autoimmunity. These findings indicate an expanded role of macrophages in SS pathology and autoimmunity, with apoptotic cells stimulating an inflammatory response in a similar way to an antiviral response through different TLRs (Table 1).

To summarize, macrophages are key modulators in the host defense mechanisms, such as the PRR signaling pathways, that are often exposed in an autoimmune context. Since these cells are essential components of the innate immune response, it is of interest to determine whether pathogenic components might trigger the autoimmune phenotype observed through PRR molecules and the impact in the antiviral response mechanisms.

#### 2.1.4. Linking Neutrophil Antiviral Response with Autoimmunity

Neutrophils are polymorphonuclear (PMN) leukocytes of the innate immune system that play a significant role in the defense against invading pathogens. They patrol the organism for signs of tissue damage or infection and participate in mediating inflammation through phagocytosis and intracellular degradation, release of granules, and NET formation after the detection of pathogenic invaders [175,176]. NETs are extracellular fibers that are composed of nuclear chromatin associated with proteins released upon neutrophil lysis. Released NETs display antimicrobial functions as they are able to capture and kill pathogens. Neutrophils may also be a source of the DNA, through NET formation, that triggers PRRs and leads to hyper-responses [177]. In lupus, DNA-containing ICs released upon NETosis are able to activate TLR9 in pDCs and induce the production of IFN-α [178]. Moreover, NET formation can trigger complement activation leading to the exacerbation of SLE pathogenesis [179]. NETs also activate neutrophils as reported by recent data through the TLR8 and TLR9 signaling cascades, although further studies need to elucidate the exact receptors and molecules involved [177,180,181]. Neutrophils are also important producers of cytokines and chemokines in order to boost the inflammatory response and recruit other immune cells at the sites of infection during viral invasion and autoimmunity [182,183].

During viral infection, neutrophils engulf virions and apoptotic cells through phagocytosis in order to eliminate viral replication and favor the clearance of the virus. NET formation can also promote viral elimination by capturing the viral particles and inactivating the virus [184,185]. Within lupus serum, ICs induce NETs and the production of type I IFNs through the activation of neutrophil Fcg receptors and TLR7 receptors [186,187]. Lood et al. [188] revealed that the TLR7/8-mediated shedding of Fc gamma receptor II A (FcgRIIA), which is the most widely expressed FcgR of the human leukocytes, shifts neutrophil function from the phagocytosis of nucleic acid-containing ICs to NETosis, a programmed form of necrosis, thus favoring their inflammatory potential while also impairing the phagocytic capacity of other immune cells, such as monocytes and DCs. They also reported that in SLE patients, FcgRIIA shedding in monocytes and neutrophils is present and correlated with the activation of neutrophils. Therefore, neutrophils seem to play an important role in regulating inflammation and autoimmunity through TLR7/8 activation and in influencing other immune cell effector functions. However, another study revealed that SLE-derived ICs activate neutrophils to release ROS and chemokines in a FcgRIIA-dependent and TLR7- and TLR9-independent manner contributing to local tissue inflammation and injury [189]. To this end, in SLE, a switch in neutrophil activity with a diminished phagocytic capacity but an increased NET formation is observed. Therefore, it remains unexplored how efficiently neutrophils will facilitate antiviral responses in an autoimmune setting (Table 1).

Taken together, these studies support the complex role of neutrophils in mediating immune responses during viral infections and autoimmune diseases. However, a direct causative link of antiviral response mechanisms of neutrophils with flares in autoimmunity remains to be established.

#### 2.1.5. Linking Natural Killer (NK) Cell Antiviral Response with Autoimmunity

Innate lymphoid cells (ILCs) are a recently discovered group of innate immune cells that originate from common lymphoid progenitors (CLPs). There are five subsets of ILCs based on the differences observed in development, phenotype, and function: NK cells, ILC1s, ILC2s, ILC3s, and lymphoid tissue-inducer (LTi) cells [190]. Natural killer (NK) cells, which belong to the family of ILCs, represent 5–20% of all human circulating lymphocyte subsets. There are two subsets of human NK cells: the CD3^−^CD56^bright^CD16^−^ and the CD3^−^CD56^dim^CD16^+^ subsets. CD56^dim^ display cytotoxic functions, whereas CD56^bright^ are important players in cytokine secretion [191]. Altered functional and regulatory profiles of NK cells could influence the outcome of several autoimmune diseases [192,193]. Upon TLR activation, NK cells produce cytokines, such as IL6, TNF-α, and (MIP)-1α, and crosstalk with other immune cells, thus having an important role in the development of inflammation and the severity of the disease. Moreover, an important role of NK cells in antiviral innate immunity has been demonstrated [194]. Upon viral infection, type I IFNs have the ability to directly regulate the activation of NK cells by promoting their proliferation and cytotoxic properties for efficient clearance of the virus [195,196].

A study by Cossu et al. [197] reported that an increase in activated CD56^bright^ NK cells with SSc progression from early to definite SSc was shown upon TLR1/2 stimulation. This increase was further combined with an enhanced secretion of IL6, TNF-α, and MIP-1α/CCL3 underlying the role of NK cells in SSc onset. CD56^+^ cells from patients at different stages of SSc respond in a different manner to TLR activation, highlighting the role of immunity in the developmental and prefibrotic SSc (Table 1). The interplay of NK cells with other immune cells, such as DCs, following TLR activation is an important homeostatic control that balances the efficiency of innate and adaptive immune responses with the risk to develop autoimmune events. Interestingly, in a study by Schuster et al. [198], it was revealed that a TRAIL^+^ NK cell subset controls immune responses during chronic murine cytomegalovirus (MCMV) infection by eliminating the effector CD4^+^ T cells thus reducing the antiviral response and hindering the clearance of the virus. In addition, they demonstrated that upon MCMV infection, mice that lack the TRAIL^+^ NK cell subset develop an autoimmune disorder that has the clinical and histopathological characteristics of SS. It is apparent that in this setting, NK cells act in a protective manner and limit autoimmune responses. This well-coordinated balance between homeostasis and chronic viral infection underlines the mechanisms of action that may culminate in systemic autoimmune phenomena with a viral etiology.

Although NK cells seem to have a significant role in the fine-tuning of antiviral response mechanisms and adaptive immune responses that may lead to autoimmune features, further studies are needed to establish their functions in the pathophysiology of autoimmune diseases and the mechanisms of action.

**Table 1:** Key findings in studies addressing PRR manifestations in innate immune cells in autoimmunity.

Cell Type	Autoimmune Context	Affected Molecule	Type of Defect/Triggering	Reference
pDCs	pSS	TLR7	Activation	[95,96,97]
pDCs	SLE	TLR7	Activation	[114,115,116]
pDCs	SLE	TLR7, TLR9	Activation (ICs stimulation, ΕΒV genome)	[118,119]
pDCs	SSc	TLR8	Activation	[122]
Monocytes	SLE	STING	Activation (mtDNA)	[130]
Monocytes	SLE	TLR7	Activation (inflamm [43])	
Monocytes	AGS	SAMHD1	SAMHD1 deficiency	[139]
Monocytes	RA	TLR2, TLR9	Activation (inflammation and EBV virions)	[144]
Monocytes	RA	TLR2	Activation (inflammation)	[146]
Monocytes	SSc	TLR8	Activation (viral EBV mRNA and proteins and inflammation)	[150]
Monocytes	SSc	MyD88, IRF7	Activation (viral EBV mRNA and proteins and inflammation)	[150]
Monocytes	pSS	TLR4, TLR5, TLR7/8	Dysregulation (inflammation)	[151]
Macrophages	SLE	AKT2	Decreased AKT2 expression (viral infection or inflammation)	[163]
Macrophages	RA	TLR2	Activation (inflammation)	[168]
Macrophages	RA	TLR2, TLR4	Activation (inflammation)	[169,170]
Macrophages	RA	TLR7	Activation (inflammation)	[171]
Macrophages	RA	TLR3, TLR7, IRF5	Activation (inflammation)	[172]
Macrophages	SS	TLR7, TLR9	Activation (inflammation)	[97,173]
Macrophages	SS	TLR7, MyD88, RSAD2, IRF7, RIG-I, MDA5	Activation (ACs and inflammation)	[174]
Neutrophils	SLE	TLR7	Activation (ICs and inflammation)	[186,187,197]
Neutrophils	SLE	TLR7/8	Activation (ICs and inflammation)	[186,187,188]
NK cells	SSc	TLR1/2	Activation (inflammation)	[188,197]

## 3. Therapeutic Manipulation of PRR Signaling in Autoimmunity

PRRs are essential elements in innate immunity and play a significant role in the host defense mechanism against viral microbes. The overactivation of PRRs or downstream molecules disrupts the homeostasis of the immune system resulting in excessive inflammatory cytokine secretion. The involvement of nucleic acid-sensing mechanisms in the immune response against infections and in autoimmune diseases makes these pathways interesting targets for drug design. The exaggerated response of the immune cells with a hyperproduction of cytokines called a “cytokine storm” plays an important role in the manifestation and progression of autoimmunity [11]. In this context, targeting the PRR signaling cascades in autoimmunity could be accomplished within the following frames: (a) blocking the binding of TLR ligands or the dimerization of the receptors and (b) interfering and inhibiting the signal transduction downstream of the PRRs [5,199].

The development of therapeutic agents for inhibiting PRR signaling, such as small molecule inhibitors, antibodies, oligonucleotides, lipid-A analogs, microRNAs, new emerging nano-inhibitors, and drugs, may help to control the hyperinflammation observed in autoimmune diseases induced by various factors, such as the uncontrolled recognition of self-nucleic acids and autoantibodies. Antimalarial drugs, such as chloroquine (CQ), hydroxychloroquine sulfate (HQ), and quinacrine, serve as antagonists for TLR7, 8, and 9 and have been used for the treatment of autoimmune diseases, such as SLE and RA. More specifically, hydroxychloroquine is a widely used antimalarial to treat both RA and SLE by modulating neutrophil function. It has recently been reported that the inhibition of TLR9 can lead to the inhibition of NET formation [200]. Antimalarial drugs are also lysosomotropic agents that target the lysosomal compartment leading to the permeabilization of the lysosomal membrane and secretion of enzymes along with signaling for apoptosis. These lysosomotropic agents could affect the PRR signaling cascades and dampen the hyper-responses to self-nucleic acids. For instance, HCQ and CQ can inhibit the uptake of nucleic acids and therefore the activation of nucleic acid sensors, such as TLR3, TLR8, TLR9, and cyclic cGAS, leading to the inhibition of the PRR-mediated activation of downstream molecules and the subsequent secretion of type I IFNs and inflammatory cytokines [201]. Another TLR7/8 inhibitor, M5049, inhibits TLR7/8 activation in pDCs, neutrophils, and monocytes, thus reducing IFN and inflammatory cytokine production. In addition, it was reported that M5049 may also block adaptive inflammation by inhibiting autoreactive B cells and seems to be a promising drug candidate [202]. Moreover, there is no evidence of inhibitors targeting CLRs and RLRs for the treatment of autoimmune diseases in clinical trials [199]. However, ARL5B, which is an MDA5-binding protein, has been reported to block the interaction of MDA5 with dsRNA [203]. In addition, DNAJB1 binds to MDA5 and prevents multimer formation attenuating type I IFN production and innate immune response [204]. In addition, another good therapeutic target could be the UNC93B1, as it has recently been demonstrated to block the cGAS–STING signaling cascade by attenuating IRF3 nuclear translocation and decreasing STING stability by promoting its intracellular degradation via the autophagy pathway [205]. Various therapeutic agents are in clinical trials targeting TLRs in order to control inflammation and hyper-responses in autoimmune diseases. This observation highlights the importance of TLRs in the initiation and progression of autoimmunity and places their agonists as ideal candidates for drug discovery in order to suppress the inflammatory response. However, the main concern of the TLR-focused effective treatment strategy should be to maintain the generic function of TLRs as they are important receptors for indicating and responding to a viral infection and other cell abnormalities of various pathology. It is of great interest to explore what happens when we suppress the response of TLRs to treat several autoimmune diseases while a viral infection occurs, as a balancing act is pivotal in order to restore homeostasis. In addition, other PRRs, such as MDA5 and STING, also play a significant role in the response to viral infections as well as autoimmunity, but their therapeutic targeting seems to be more challenging than anticipated. Attenuating STING and other PRR activities, which are important regulators of the immune response to ameliorate inflammation in the context of autoimmunity, may give rise to new challenges in order to combat viral infection.

Several molecules have a significant role in the signal transduction downstream of PRRs, including MyD88, IRAK4, TRAF6, TAK1, TRIF, TBK1, and NF-κB, as mentioned previously. The development of inhibitors targeting these molecules is of great importance in order to treat autoimmune diseases. Three IRAK4 inhibitors have entered clinical trials with promising results for the treatment of RA [206,207]. Many studies use mouse models in order to explore the treatment efficiency of the inhibitors. For example, polyphyllin I (PPI) is an NF-κB inhibitor, as it diminished the phosphorylation of the NF-κB subunit p65 and the subsequent p65 accumulation in the nucleus. It was revealed that PPI seem to ameliorate synovial inflammation by suppressing the NF-κB-induced inflammatory signaling observed in macrophages in an RA mouse model [208]. Taraxasterol (TAR) diminished IL-1β-induced synovial inflammation in human fibroblast-like synoviocytes RA (HFLS-RA) in vitro and the progression of the disease in a RA mouse model in vivo. TAR seems to be a considerable therapeutic compound for RA by suppressing the NF-κB and NLRP3 inflammasome-induced synovial inflammation [209]. In addition, the role of IRFs in the regulation of the immune response makes these transcriptional regulators important therapeutic candidates for drug discovery [210]. Recently, it was reported by Li et al. [211] that dysregulated IRF5 activity is a driver of SLE disease onset and severity. Preclinical treatment of NZBWF1 mice with an IRF5 inhibitor led to reduced antinuclear autoantibodies, dsDNA titers, and circulating plasma cells and attenuated SLE pathology to improve survival. In ex vivo human studies, the inhibitor blocked SLE serum-induced IRF5 activation and reversed basal IRF5 hyperactivation in SLE immune cells [212].

To conclude, chemical agents targeting several TLRs and downstream effector molecules and cytokines offer novel opportunities for the prevention of or intervention against virus-induced infectious diseases and autoimmunity. Targeting PRRs with drugs may also induce harmful immune activation or unwanted immunosuppression dampening antiviral responses. From this perspective, targeting selective innate immune cell types, such as monocytes, macrophages, DCs, or neutrophils, that play critical roles in host defense against viral compounds could result in improved therapeutic outcomes in the treatment of viral infection and autoimmunity.

## 4. Synthesis, Concluding Remarks, and Open Questions

In this review, we discussed the effects of an excess or a deficiency of PRR signaling in autoimmune diseases and its relation with viral infection, focusing on the cells of the innate immune arm, such as monocytes, macrophages, DCs, and neutrophils, and the cells that bridge the innate with adaptive immune responses, such as NK cells. In autoimmune diseases, aberrant cytokine production, tissue damage, and hyperinflammation inflammation along with the genetic predisposition and environmental factors influence the function of PRRs and the expression of downstream molecules. The aberrant innate immune response influences the adaptive immune cells leading to a disrupted antiviral response and increasing the risk for autoimmunity or disease flares when an autoimmune disease is already present. Further research is needed to expand our knowledge regarding the influence of abnormal PRR signaling in the pathogenesis of autoimmune diseases.

Over the last years, the emerging role of antiviral responses in innate immune cells during autoimmune manifestations has posed new challenges and questions about the host defense mechanisms. To provide insight into the PRR cascade in innate immune cell inflammatory response in autoimmunity, it is crucial to determine whether PRR alterations drive or exaggerate autoreactive phenotype (e.g., inflammatory cytokine production, interplay with adaptive immune cells to promote NET formation, and autoantibody production) and if these signals imprint on the inflammatory cascade. Moreover, it is of interest to determine whether the currently used therapeutic agents or drug candidates for the treatment of autoimmune disorders may deregulate the antiviral response in innate immune cells. Regarding the targeting of TLRs for the treatment of autoimmunity, it is essential to preserve their physiological function in the context of viral immunity while ameliorating their effects on autoimmune responses. In this context, it is of great importance to delineate whether the antiviral and/or inflammatory response pathways that are activated differ during systemic autoimmunity as compared to organ-specific autoimmunity. Modulating specific PRR activity in innate immune cells in patients suffering from such PRR-dependent autoimmune diseases, such as SLE, RA, SSc, and Sjogren’s syndrome, may be of therapeutic value. To accomplish that, it is essential to focus on the discovery of inhibitors or other molecules that could efficiently modulate the immune response and with reduced off-target effects. Addressing such questions may revolutionize therapeutic approaches for autoimmune diseases.

## Data Availability

Not applicable.
